# A Shocking Spiral: Methamphetamine-Induced Electrical Storm in End-Stage Cardiomyopathy

**DOI:** 10.7759/cureus.89507

**Published:** 2025-08-06

**Authors:** Stephen T Aben, Karim Akl, Kumar Akshay

**Affiliations:** 1 Department of Internal Medicine, Jersey City Medical Center, Jersey City, USA; 2 Department of Pulmonary and Critical Care Medicine, Jersey City Medical Center, Jersey City, USA

**Keywords:** cardiomyopathy, electrical storm, extracorporeal membrane oxygenation (ecmo), methamphetamine, substance-induced arrhythmia, ventricular tachycardia

## Abstract

An electrical storm (ES) represents one of cardiology's most formidable and life-threatening crises, marked by relentless ventricular arrhythmias within a 24-hour period. While stimulant cardiotoxicity is an escalating concern, the devastating role of methamphetamine in triggering refractory ES and its deleterious outcomes in advanced cardiomyopathy, particularly within the critical care setting, remains profoundly underreported and poorly understood. We present the urgent case of a 44-year-old male with end-stage dilated cardiomyopathy and chronic, heavy methamphetamine abuse, who spiraled into incessant ventricular tachycardia (VT) storm following acute methamphetamine use. Despite aggressive anti-arrhythmic therapy including over 35 defibrillation shocks, he developed profound cardiogenic shock, intractable arrhythmias, and a rapid progression to multi-organ failure. Maximal support with both extracorporeal membrane oxygenation (ECMO) and intra-aortic balloon pump (IABP) was required, alongside continuous renal replacement therapy (CRRT) for escalating hyperkalemia and renal failure. Despite heroic multidisciplinary efforts, his course was complicated by recurring sepsis and ultimately culminated in progressive multi-organ dysfunction, leading to withdrawal of care on hospital day 16. This critical case study illuminates the therapeutic challenges posed by methamphetamine-induced VT storm in advanced cardiomyopathy. It vividly underscores the imperative for immediate recognition, rapid initiation of a multidisciplinary approach, and aggressive supportive care for such catastrophic arrhythmogenic crises. Furthermore, it highlights the devastating, often irreversible synergy between stimulant cardiotoxicity and structural heart disease, demanding urgent awareness among clinicians to mitigate this growing public health emergency.

## Introduction

An electrical storm (ES), defined as three or more episodes of sustained ventricular tachycardia (VT) or ventricular fibrillation (VF) within a 24-hour period, each separated by at least five minutes, is a medical emergency characterized by heightened electrical instability of the myocardium [1,2]. In the critical care setting, ES presents unique therapeutic challenges due to the frequent coexistence of advanced cardiomyopathy, hemodynamic fragility, and multiorgan dysfunction [3]. Mortality remains high, with predictors including low left ventricular ejection fraction (LVEF), right ventricular dysfunction, and the need for vasopressors at presentation [3]. Furthermore, VT clusters are not only a manifestation of malignant arrhythmia burden but also serve as an ominous prognostic marker in patients with structural heart disease, particularly those with dilated cardiomyopathy [4].

While ischemic cardiomyopathy and inherited arrhythmic syndromes are the predominant substrates for ES, stimulant-induced cardiotoxicity is an increasingly recognized but underappreciated trigger [5]. Methamphetamine and related compounds now represent the second most commonly abused illicit substances worldwide, with epidemiologic data indicating a rising incidence of associated cardiovascular pathology, including heart failure and sudden cardiac death [6,7]. Chronic use is associated with a distinct form of nonischemic cardiomyopathy-methamphetamine-associated cardiomyopathy (MAC), characterized by biventricular dysfunction, arrhythmia susceptibility, and poor reversibility in late-stage presentations [6].

We report a critical case of a 44-year-old man with end-stage non-ischemic dilated cardiomyopathy and chronic heavy daily methamphetamine use who developed a refractory ES shortly after methamphetamine consumption. Despite aggressive antiarrhythmic therapy and full mechanical circulatory support, the clinical course was rapidly complicated by hemodynamic collapse and multi-organ failure. 

## Case presentation

A 44-year-old male with a known history of non-ischemic dilated cardiomyopathy was brought to the emergency department by emergency medical services for the evaluation of sudden-onset palpitations, dyspnea, and confusion. According to his family, the onset of symptoms occurred shortly after a witnessed episode of methamphetamine use. Besides a history of chronic heavy methamphetamine use complicated by established dilated cardiomyopathy with a previously documented ejection fraction of 30% and severe mitral regurgitation, the patient had no other chronic medical conditions. Prior comprehensive evaluation, including coronary angiography, transesophageal echocardiography, and cardiac MRI, revealed no alternative etiologies for his heart failure. Of note, the patient was non-adherent to guideline-directed medical therapy for heart failure and had no medical insurance. The patient had been referred multiple times for peer recovery support; however, he continued to engage in heavy daily methamphetamine use. 

**Figure 1:**
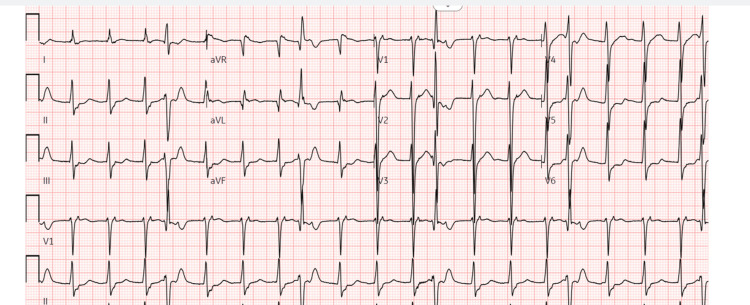
12-lead electrocardiogram on presentation showing a regular narrow-complex tachycardia at a rate of 121 beats/min interpreted as SVT SVT: supraventricular tachycardia

Upon arrival to the emergency department, the patient was somnolent, hypotensive (blood pressure: 90/54 mmHg), and tachycardic (heart rate: 150 bpm). He was promptly intubated for worsening mental status. Physical examination was notable for jugular venous distension, a grade IV systolic murmur at the apex, bilateral diffuse rales, and 2+ pitting edema in both lower extremities. Initial investigations revealed a positive urine toxicology screen for methamphetamines and tetrahydrocannabinol, a BNP of 738 pg/mL, high-sensitivity troponin of 28 ng/L, serum glucose of 279 mg/dL, creatinine of 1.1 mg/dl, BUN of 28, sodium of 134, and potassium of 7.1 mmol/L. Liver enzymes were borderline elevated with alanine transaminase (ALT) of 41 U/L and aspartate transaminase (AST) of 39 U/L. A respiratory viral panel was positive for both Influenza A and RSV. Empiric broad-spectrum antibiotics with ceftriaxone and doxycycline were administered for presumed pneumonia-related sepsis, and oseltamivir was initiated for the treatment of influenza. Electrocardiogram showed a narrow-complex tachycardia without discernible P waves, consistent with atrioventricular nodal reentrant tachycardia (AVNRT) (Figure 1).

He underwent a single synchronized direct-current cardioversion and successfully reverted to normal sinus rhythm. Hyperkalemia was appropriately managed, with repeat potassium level returning at 4.0 mmol/L. The patient was started on a titratable norepinephrine infusion for hemodynamic support and initiated on intravenous furosemide. He was subsequently transferred to the intensive care unit (ICU) for further management.

**Figure 2:**
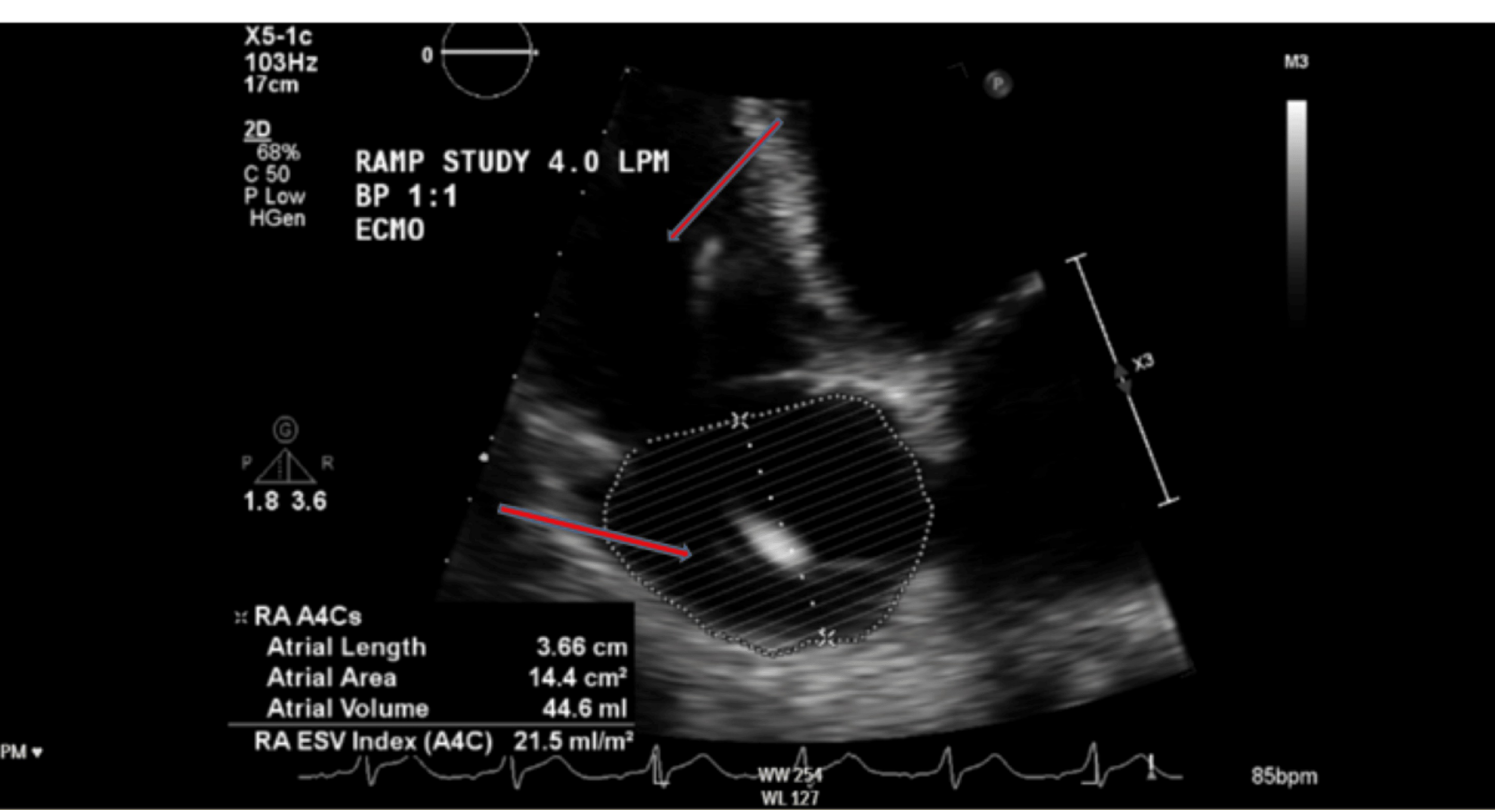
TTE apical four-chamber view showing a markedly dilated right atrium and ventricle TTE: transthoracic echocardiogram

**Figure 3:**
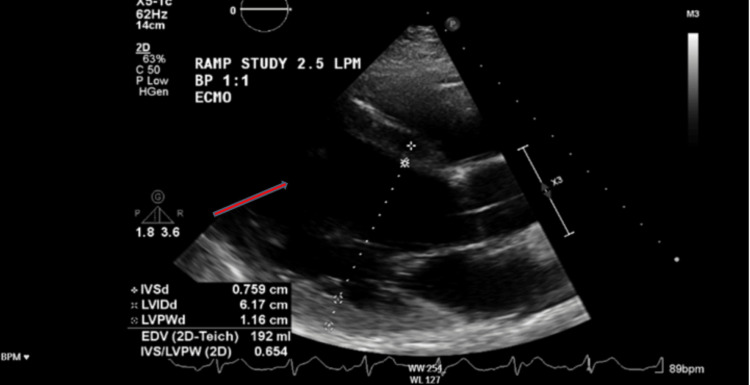
TTE parasternal long-axis view showing a severely dilated left ventricle TTE: transthoracic echocardiogram

**Figure 4:**
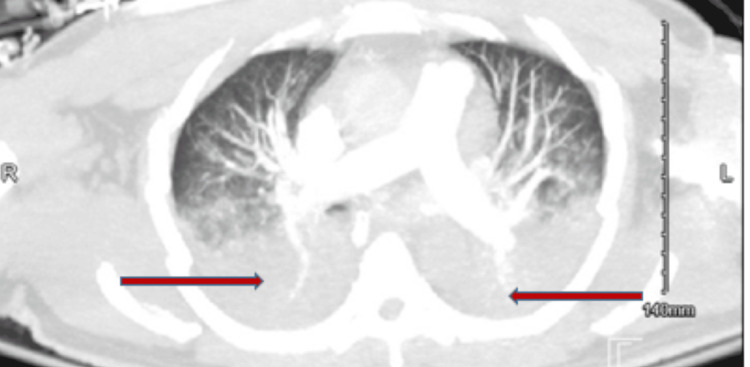
CT of the chest showing diffuse bilateral dense pulmonary infiltrates consistent with acute pulmonary edema and possible pneumonia CT: computed tomography

 Echocardiography demonstrated biventricular failure with an ejection fraction of 25% (Figures 2-3, Video 1) and severe mitral regurgitation (Video 2). Swan-Ganz catheter confirmed cardiogenic shock with pulmonary hypertension (pulmonary capillary wedge pressure (PCWP) 27 mmHg, pulmonary artery pressure (PAP) 60/30 (40) mmHg, right atrial pressure (RAP) 17 mmHg, and central venous pressure (CVP) 21 mmHg). Meanwhile, his chest CT scan revealed dense bilateral infiltrates consistent with pneumonia and/or pulmonary edema (Figure 4).

**Video 1:** TTE apical four-chamber view showing severely dilated right atrium and left ventricle with globally reduced left ventricular systolic function (EF ~25%). The video highlights the severe biventricular dilation and poor contractility characteristic of methamphetamine-induced cardiomyopathy. TTE: transthoracic echocardiogram

**Video 2:** TTE parasternal long-axis view demonstrating severe mitral regurgitation with a large regurgitant jet directed posteriorly into a markedly dilated left atrium. The clip highlights the severe regurgitant flow and chamber dilatation in the setting of end-stage methamphetamine cardiomyopathy TTE: transthoracic echocardiogram

**Figure 5:**
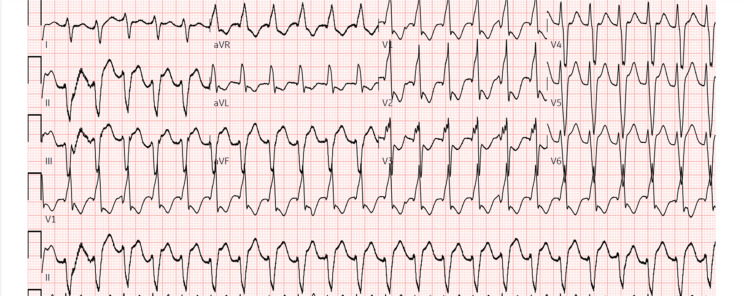
12-lead electrocardiogram showing sustained monomorphic ventricular tachycardia episodes with the right bundle branch block, resulting in multiple synchronized cardioversions (a total of 35 shocks delivered)

Shortly after arriving in the ICU, the patient developed incessant ventricular tachycardia (Figure 5), refractory to multiple anti-arrhythmic agents including amiodarone, lidocaine, and procainamide, as well as over 35 defibrillation shocks. The arrhythmia culminated in high-grade atrioventricular block, necessitating transvenous pacing. Given worsening hemodynamic instability and respiratory failure, intra-aortic balloon pump (IABP) support was inserted in the left common femoral artery, and the patient was cannulated for venoarterial extracorporeal membrane oxygenation (VA-ECMO) at the bedside. VA-ECMO was achieved using a right superficial femoral artery 6F distal reperfusion cannula, a right common femoral artery 21F Femflex reinfusion cannula, and a right common femoral vein 25F multiport Biomedicus drainage cannula. Subsequent interval blood work demonstrated a progressively worsening metabolic profile, including lactic acidosis with levels ranging from 5.5 to 11.6 mmol/L, persistent hyperkalemia ranging from 6.6 to 7.7 mmol/L refractory to standard therapy, and acute kidney injury with creatinine levels increasing from 2.2 to 3.03 mg/dL. Continuous renal replacement therapy (CRRT) was started for hyperkalemia and progressive renal failure.

The patient's hospital course was further complicated by gastrointestinal bleed, coagulopathy, recurring sepsis, and arrhythmias. Despite temporary hemodynamic stabilization, the patient progressed to multi-organ failure with suspected anoxic brain injury. After multidisciplinary discussions and family consultation, care was withdrawn, and the patient expired on hospital day 16.

## Discussion

An ES is a highly malignant manifestation of ventricular arrhythmia, particularly in patients with structural heart disease, and is associated with substantial morbidity and mortality. In this context, methamphetamine use serves as a potent pro-arrhythmic trigger, promoting ventricular arrhythmias through sympathetic overstimulation, direct myocardial toxicity, and progressive myocardial fibrosis [7,8]. Although prior case reports have documented methamphetamine-associated cardiomyopathy presenting as complex ventricular arrhythmias [9-11], reports detailing full-blown ES mechanical circulatory support in critically ill patients remain exceedingly rare. This case highlights a catastrophic convergence of end-stage cardiomyopathy and stimulant toxicity, culminating in incessant ventricular tachyarrhythmias unresponsive to multiple antiarrhythmic strategies and mechanical circulatory support. In such cases, ES may reflect not only arrhythmic instability but irreversible myocardial and systemic decompensation.

The etiology of ES is multifactorial, often precipitated by drug toxicity, electrolyte abnormalities, acute heart failure, ischemia, thyrotoxicosis, and infections [12]. Advanced age, male sex, and chronic kidney disease further heighten the risk [13]. Although it remains unclear whether ES correlates directly with poor outcomes or is simply an epiphenomenon of advanced heart failure, our patient potentially had identifiable triggers for ES. However, these triggers were not clinically apparent until after the onset of ES episodes. Nevertheless, even when identifiable triggers are present, sustained ventricular tachycardia (VT) seldom occurs without a predisposing vulnerable myocardial substrate, as demonstrated in this case [2]. The development of ES in patients with structural heart disease, such as dilated cardiomyopathy, has been shown to be an independent predictor of poor long-term survival, even in the presence of ICDs [14], which are otherwise protective against sudden arrhythmic death. This underscores the fact that ES is not merely a transient arrhythmic event but reflects advanced disease substrate and systemic vulnerability, particularly in cases like ours involving methamphetamine-induced cardiotoxicity.

ES confers a substantial risk of recurrent ventricular arrhythmias and death, yet evidence consistently demonstrates that successful acute intervention, particularly through catheter ablation, is associated with significantly lower rates of VT recurrence and enhanced one-year survival [15]. Although catheter ablation and sympathetic denervation are established first-line therapies for refractory VT, our patient’s profound hemodynamic instability necessitated emergent initiation of mechanical circulatory support. Beyond its hemodynamic effects, IABP counterpulsation has been reported to acutely suppress VT storm by modulating ventricular loading conditions [16]. However, the coexistence of pulmonary hypertension, as seen in this case, is associated with particularly poor outcomes in methamphetamine-induced cardiomyopathy [17,18]. Despite temporary stabilization with ECMO and IABP, the patient’s recovery was ultimately limited by refractory multi-organ failure.

## Conclusions

Methamphetamine-induced VT storm in advanced cardiomyopathy is a catastrophic event with limited therapeutic options and poor prognosis despite aggressive management. This case significantly contributes to the limited literature on methamphetamine-associated ES in critically ill patients, underscoring the need for early recognition and aggressive interventions. It highlights the catastrophic synergy between stimulant toxicity and structural heart disease, offering valuable lessons for electrophysiologists, intensivists, emergency care physicians, and heart failure specialists in developing tailored, multidisciplinary approaches to manage such complex arrhythmogenic crises. Furthermore, it draws attention to the escalating public health crisis posed by methamphetamine use, particularly its frequently underrecognized cardiotoxic effects, which necessitate heightened clinical vigilance and broader preventive efforts.
